# Case report: Early-onset Parkinson's disease with initial spastic paraparesis and hyperreflexia caused by compound heterozygous *PRKN*-gene exon 2 and 4 deletions

**DOI:** 10.3389/fneur.2022.969232

**Published:** 2022-11-17

**Authors:** Ida Jensen, Corinna Hendrich, Martin Klietz, Georg Berding, Günter U. Höglinger, Florian Wegner

**Affiliations:** ^1^Department of Neurology, Hannover Medical School, Hannover, Germany; ^2^Department of Human Genetics, Hannover Medical School, Hannover, Germany; ^3^Department of Nuclear Medicine, Hannover Medical School, Hannover, Germany

**Keywords:** *PRKN*-gene, Parkin, *PRKN2*, young onset parkinsonism, early onset Parkinson's disease (EOPD), exon 2, exon 4, case report

## Abstract

Pathogenic variants in the Parkin-gene (*PRKN*) are among the most common genetic causes of early onset Parkinson's disease (EOPD). Patients with EOPD can present with atypical clinical features and misdiagnosis is frequent. Here, we report a clinical phenotype with atypical signs and symptoms of a 35-year-old male patient with EOPD caused by a compound heterozygous *PRKN*-gene deletion of exons 2 and 4. After the initial diagnosis of stiff person syndrome, the patient was admitted to our department for a second opinion after 8 years of untreated disease progression. The patient presented with prominent spastic paraparesis pronounced on the right side and hyperreflexia as well as Parkinsonism with rigidity predominantly affecting the upper limbs, bradykinesia, and resting tremor. In the diagnostic assessment, magnetic evoked potentials to the anterior tibial muscles showed a low amplitude on the right side, compatible with pyramidal tract disturbance. However, an MRI of the head and the spine did not show any pathologies or atrophy. A [^123^I] FP-CIT SPECT scan revealed profoundly and left-pronounced reduced striatal uptake suggesting a neurodegenerative Parkinson's syndrome. Even though an acute levodopa challenge did not show marked improvement of symptoms, the chronic levodopa challenge with up to 450 mg/day significantly reduced the rigidity and bradykinesia. Surprisingly, spastic paraparesis and hyperreflexia diminished under dopaminergic treatment. Finally, genetic analysis by next-generation sequencing *via* copy number variant analysis (CNV) and multiplex ligation-dependent probe amplification (MLPA) confirmed compound heterozygous deletions of exons 2 and 4 in the *PRKN*-gene. As presented in this case, the awareness of atypical clinical symptoms of EOPD is essential to prevent misdiagnosis in young patients.

## Introduction

EOPD describes the early onset of parkinsonism under the age of 40 years (1). About 3–7% of all cases of Parkinson's disease (PD) in the Western world are classical EOPD (2–5). Homozygous or compound heterozygous pathogenic variants in the *PRKN*-gene, also known as Parkin or *PARK2*, are among the most common genetic causes of EOPD. They can be detected in 6–12% of PD cases with an onset before the age of 50 years and 30% of cases with an onset before the age of 30 years (6–8). The *PRKN* gene is ~1.38 Mb long and one of the largest genes in the human genome. The protein product of the *PRKN*-gene is a 465-amino-acid E3 ubiquitin ligase that mediates mitochondrial intracellular Ca^2+^ homeostasis, adaptation to stress, and cell death (9–11). Pathogenic variants of the *PRKN-*gene have been described across all of its 12 exons (9). They are highly variable, including exonic rearrangement (deletion or duplication), small insertions, or point mutations (6, 12–15). They can be found in heterozygous (only one allele affected), compound heterozygous (both alleles affected by different mutations), or homozygous (both alleles affected by the same mutation) states (16–19). The average age of onset of patients with only one allele affected is about 40 years, while it is about 30 years in patients with mutations in both alleles (20). In general, patients with EOPD not only present with typical PD symptoms, such as bradykinesia, tremor, and rigor, but also with atypical symptoms such as dystonia and pyramidal signs (6). Some patients report an improvement in their symptoms after sleep (1). Concerning non-motor symptoms, depression and anxiety are frequent, while the incidence of cognitive impairment is low (14, 21–24). Patients usually show a slower disease progression than those with idiopathic PD and a remarkable improvement of symptoms in response to low-dose levodopa therapy. However, after long-term treatment, they also exhibit earlier motor complications such as dyskinesia or motor fluctuations (25). A specific genotype-phenotype correlation of *PRKN*-gene pathogenic variants has not been established yet as the symptoms vary substantially. Because of those clinical variations, delays in the EOPD diagnosis are commonly leading to significant deceleration in the treatment and genetic counseling of patients and their families.

Therefore, it is essential to expand the knowledge of different *PRKN* variants and their specific phenotypes. In the following case, we report atypical signs and symptoms as well as imaging features and treatment response of a 35-year-old male patient with EOPD caused by a compound heterozygous *PRKN*-gene deletion of exons 2 and 4. This case report aims to highlight the awareness of atypical symptoms in EOPD with particular pathogenic variants of the *PRKN*-gene which can prevent patients from misdiagnosis and lead to earlier sufficient treatment.

## Case description

### Medical history

The formerly healthy Caucasian patient reported an onset of symptoms at the age of 28 years. The patient recognized a progressing stiffness of the lower limbs, accompanied by muscle pain, especially in the lower back and lower limbs. He further recognized an increased tendency for muscle cramps and spasms after physical strain. Over the next 5 years, the stiffness of the lower limbs increased, leading to a broad-based gait. The patient felt exhausted by walking distances over 500 m and adapted his daily activities to avoid walking. He consulted a neurologist in 2018. However, no clear diagnosis explaining the symptoms was found and a stiff person syndrome was assumed.

In the following 3 years, a right-sided tremor, rigidity, and impairment of fine motor skills were added to the symptoms. The patient reported an improvement in motor symptoms through sleep. However, he generally recognized decreasing sleeping quality, increasing anxiety, and depressive symptoms.

**Figure 1:**
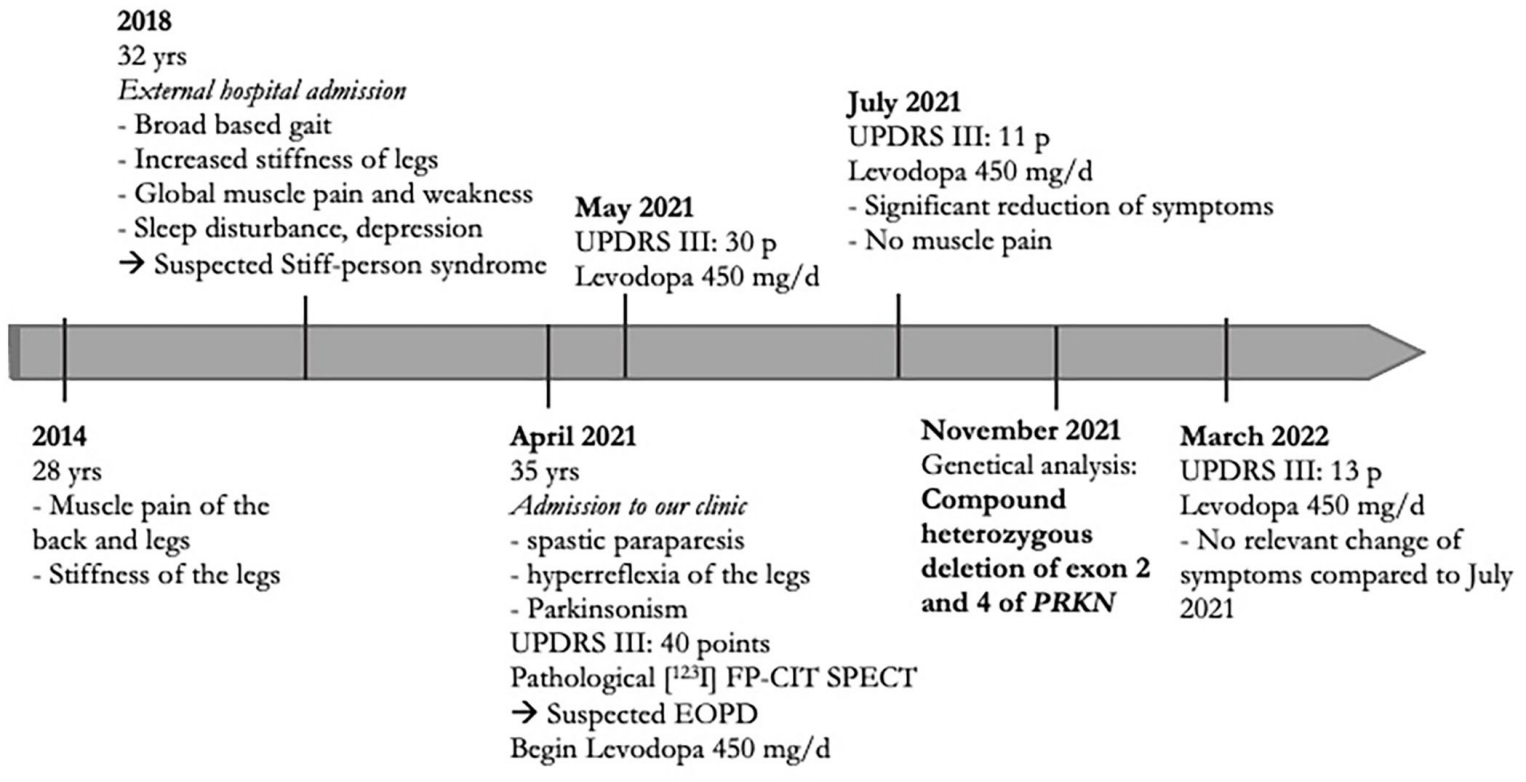
Timeline with relevant anamnestic data.

Concerning the patient's family history, no neurological disorders were reported. Consanguinity did not occur in his family. At the age of 35 years, the patient visited our outpatient clinic with a progressing gait disturbance and muscular pain which led to admission (for the timeline of symptoms, see Figure 1).

### Clinical findings

The physical examination revealed a prominent spastic paraparesis pronounced on the right side and hyperreflexia of the lower limbs. The patient presented a spastic broad-based gait pattern without signs of sensory ataxia, accompanied by muscle pain in the lower limbs and back. Furthermore, he showed Parkinsonism including arm-accentuated rigor with cogwheel rigidity and a right-pronounced resting-, action-, and postural tremor. A mild hypomimia was apparent but no speech problems occurred. Fine motor skills like finger-tapping or hand movements displayed an amplitude decrement and deceleration midway through the task.

### Diagnostic assessment

Extensive diagnostic tests were performed to rule out several differential diagnoses that can present as Parkinson's disease look-alikes with early onset (26, 27). The serology of our patient only revealed a mild reduction of vitamin D and folate. A lumbar puncture showed an average leukocyte count (1.3 cells/μl) with normal protein (0.43 g/l) and lactate (1.40 mmol/l). No specific intrathecal production of IgG was detected. The examination of autoimmune antibodies in serum and CSF remained negative, and diagnostics concerning M. Wilson remained negative. Nerve conduction and electromyography were unremarkable regarding a previously suspected stiff person syndrome. Interestingly, magnetic-evoked potentials to the anterior tibial muscles displayed a low amplitude on the right side, compatible with pyramidal tract disturbance. An MRI of the head and the spine did not detect any pathologies or pronounced atrophy.

The patient scored 40 points in the MDS-UPDRS III but did not show any immediate improvement of symptoms after an acute levodopa challenge, as he still scored 40 points in the “on”-state. Nevertheless, tremor analysis revealed a tremor frequency of 6.7 Hz, compatible with Parkinson's disease tremor. Montreal Cognitive Assessment (MoCA, German version) and extensive neuropsychiatric testing (Consortium to Establish a Registry for Alzheimer's Disease, CERAD) were normal (MoCA: 30/30 points; CERAD, z = −1.0). Concerning autonomic dysregulation, bladder sonography and a Schellong-test for circulatory function were unremarkable.

**Figure 2:**
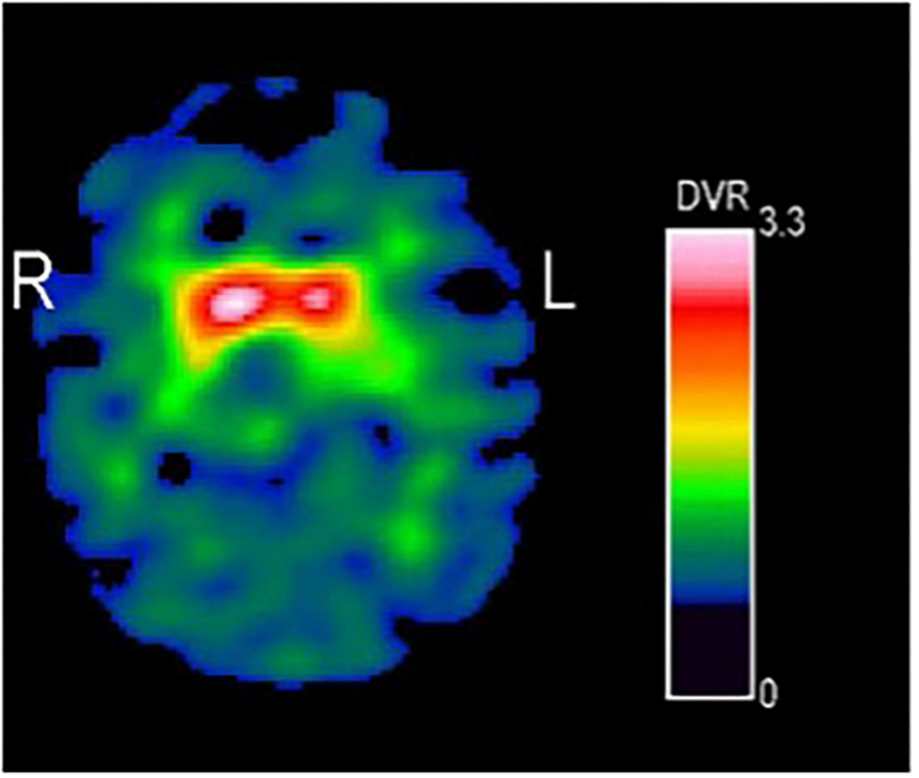
[^123^I] FP-CIT-SPECT of our EOPD patient showed profoundly reduced binding at dopamine transporters on nigrostriatal neurons. Reductions were slightly more pronounced on the left side for the caudate nucleus (right: −41%; left: −48%) and the putamen (right: −67%; left: −71%) as compared to the age-related reference (DaTQUANT^TM^ software, GE Healthcare).

[^123^I]*N*-ω-fluoropropyl-2β-carbomethoxy-3β-{4-iodophenyl}nortropane (FP-CIT) SPECT (Single-photon emission computed tomography) revealed profoundly and left-pronounced reduced striatal uptake at presynaptic dopamine transporters, suggesting a neurodegenerative Parkinson syndrome (Figure 2). [^18^F]FDG-PET's search of the brain for signs of an atypical Parkinson's syndrome remained unremarkable. In particular, no significant reduction of glucose metabolism was detected in the striatum based on visual assessment and statistical parametric mapping.

The young patient presenting with Parkinsonism and atypical symptoms (hyperreflexia and spastic paraparesis) and the pathological [^123^I] FP-CIT-SPECT led us to analyze EOPD-genes associated with spasms and hyperreflexia as atypical symptoms.

### Genetic analysis

Genomic DNA was extracted from EDTA blood samples. DNA enrichment and library preparation were performed using the xGen Exome V02, IDT (Integrated DNA Technologies, Inc., Coralville, Iowa). Whole-exome sequencing (WES) was performed on an Illumina NextSeq 500 using the NextSeq 500/550 High Output v2 kit (Illumina, San Diego, California). Alignment to the reference genome build (GRCh37) was performed using megSAP, version 0.1-710-g52d2b0c (Institute of Medical Genetics and Applied Genomics, University of Tübingen, Germany). Variant prioritization and visualization were performed with GSvar, version 2018_04, and with Alamut visual, version 2.11 (Interactive Biosoftware, Rouen, France). Variants were classified according to the criteria proposed by the American College of Medical Genetics and Genomics (ACMG) and the genes *ATP13A2, DNAJC6, FBXO7, GBA, GCH1, LRRK2, PARK7, PINK1, SNCA, SPG11, VPS13C*, and *PRKN* including flanking intronic regions (at least-3 up to +8 bp) were analyzed. Additionally, *PRKN, PARK7, SNCA, PINK1*, and *GBA* were analyzed by multiplex ligation-dependent probe amplification (MRC-Holland) for deletions or duplications. The examination was performed according to the protocol (Kit P051, MRC-Holland, Amsterdam) with fragment analysis in a Beckman Coulter Sequencer GeXP. An evaluation was performed with the software Sequence pilot (JSI medical systems).

Genetic analysis by next-generation sequencing including copy number variant analysis (CNV) by using ClinCNV, Version 1.16.6, showed compound heterozygous deletions of exon 2 (NM_004562.3: c.(7+1_8–1)_(171+1_172–1)del p.? and exon 4 (NM_004562.3: c.(412+1_413–1)_(534+1_535–1)del.p? in the *PRKN*-gene indicating genetic Parkinson's disease. Both deletions were confirmed by MLPA. Subsequently, a complementary MLPA of the patient's parents was conducted. Both did not show any symptoms of a movement disorder at the age of 62 years. The MLPA revealed the heterozygous deletion of *PRKN* exon 2 of the patient's mother and the heterozygous deletion of *PRKN* exon 4 of his father. As both parents carried one variant of the deletions, compound heterozygosity in the patient could be confirmed.

According to the actual ACMG Standards and Guidelines, deletions of exons 2 and 4 were rated as “pathogenic” (class 5) (28). The gene-specific MDSGene database lists deletions of exon 2 as pathogenic. Deletions of exon 4 are listed as probably pathogenic and documented in combination with EOPD in several patients (25, 29–32).

### Therapeutic intervention

After the [^123^I] FP-CIT-SPECT had revealed nigrostriatal degeneration, we prescribed low-dose levodopa/carbidopa (125 mg three times a day) and recommended increasing the treatment up to 450 mg/day. Other treatment options such as dopamine receptor agonists were previously discussed but rejected by the patient. Additionally, amitriptyline (75 mg/day) was added to reduce depressive symptoms and anxiety. We also recommended a comprehensive rehabilitation program and Parkinson's syndrome training.

### Follow-up and outcome

A follow-up in May 2021 at age 35 showed a significant improvement in all symptoms under treatment with 450 mg/day levodopa. He had recognized a further decrease in rigidity, stiffness, and muscle pain. His tremor was almost gone. The patient was able to walk long distances of about 3,000 m.

Furthermore, his sleep quality subjectively improved. Clinically, the patient scored 11 points in the MDS-UPDRS III in the “on”-state, a significant decrease of 72.5% compared to the beginning of treatment. Surprisingly, the spastic paraparesis and hyperreflexia also diminished under dopaminergic treatment. The tendon reflexes of the lower limbs were weak and no spasm in the lower limbs could be detected anymore. Concerning complications, the patient presented with mild dyskinesia of the trunk as a side effect of levodopa treatment. His gait was fluent and less broad-based with a residual spastic component. He stated that he had already tried a higher dosage of levodopa (600 mg/day) but had to stop because dyskinesia got worse and disrupted his daily activities.

The patient presented himself again at the age of 36 years, in the middle of March 2022. He reported that his symptoms and improvement under treatment with 450 mg/day levodopa remained stable. He scored 13 points in the MDS-UPDRS III. No cognitive decline or new symptoms were reported.

## Discussion

We present a case of a 35-year-old patient with compound heterozygous exons 2 and 4 deletions in *PRKN* and uncommon clinical manifestation.

Unfortunately, many patients with EOPD remain undiagnosed for a long time due to atypical clinical features. This patient was initially diagnosed with stiff person syndrome due to spastic paraparesis and brisk reflexes. Over disease progression, the patient developed classical parkinsonian features which gave a clue for an EOPD and led to the diagnostic pathway.

A specific genotype-phenotype correlation of patients with pathogenic variants in the *PRKN* gene has not been established yet. The correlations between phenotype and genotype are uncertain, as reported by Kasten et al., who investigated 958 Parkin mutation carriers of 663 families. Additionally, the authors described a huge proportion of non-reported phenotypic features, which complicates further definitions (25). Lücking et al. examined 73 families with EOPD. Among those, 36 families (49%) presented with *PRKN*-gene variants. Especially structural variants such as deletions and duplications, and also single nucleotide variants, were described (6). Those patients were more likely to have dystonia, symmetric involvement, and hyperreflexia. However, those symptoms tended to vary (6). Atypical symptoms, as seen in our patient, were described in other case reports of patients with *PRKN*-variants, especially Asian patients (30–32). However, our patient did not mention any Asian relatives. In other publications, clinical characteristics of North African and European patients with pathogenic *PRKN*-gene variants were indistinguishable from those of patients with idiopathic Parkinson's disease (33, 34).

The patient mentioned an improvement in his symptoms after sleep which was reported in association with pathogenic *PRKN*-gene variants before (6). Concerning non-motor symptoms, the patient suffered from depression that was described concerning EOPD (6). No cognitive decline or dementia was discovered which is in line with previously published cases (6). The substantia nigra and, to a lesser extent, the locus coeruleus seem to be more selectively affected in patients with *PRKN* mutations compared to patients with idiopathic Parkinson's disease (6).

The yet unclear genotype-phenotype correlation highlights the diagnostic value of genetic testing for correct diagnosis and prediction of patients' prognosis. Both CNV analysis and MLPA used in our genetic analysis revealed compound heterozygous deletions of exons 2 and 4 in *PRKN*. Those deletions lead to a frameshift and consequently to a premature stop codon. However, even though few case reports with similar pathogenic variants were described, the clinical phenotype of those cases remained largely uninformative which hinders a direct comparison.

The parkinsonian symptoms of our patient improved significantly after constant levodopa treatment which is frequent in EOPD (25). Surprisingly, the paraparesis and hyperreflexia were also not recognizable anymore after 5 weeks of treatment and the positive effect remained constant over 1 year. To our knowledge, this has not been described in a case before.

The patient reported that he could not further increase treatment over the dose of 450 mg levodopa/day due to dyskinesia. Early levodopa-associated dyskinesias in EOPD are well-known and can complicate treatment, even in minimal doses of levodopa (6, 9, 35). However, the patient was relieved because of the successful levodopa treatment and felt nearly as good as 8 years ago before his symptoms began.

In conclusion, we identified pathogenic compound heterozygous deletions of exons 2 and 4 in the *PRKN* gene in a 35-year-old patient with EOPD. The patient presented with an atypical phenotype and Parkinsonism. As presented in this case, the awareness of atypical clinical symptoms of EOPD, such as spastic paresis, is essential to prevent misdiagnosis in young patients. All of the patient's symptoms improved under oral levodopa therapy, highlighting the importance of deep phenotyping and early diagnosis for adequate treatment.

## Data availability statement

The datasets presented in this article are not readily available because of ethical and privacy restrictions. Requests to access the datasets should be directed to the corresponding author.

## Ethics statement

Ethical review and approval was not required for the study on human participants in accordance with the local legislation and institutional requirements. The patient provided his written informed consent to participate in the study. Written informed consent was obtained from the individual(s) for the publication of any potentially identifiable images or data included in this article.

## Author contributions

IJ, MK, and FW contributed to clinical assessment. CH contributed to genetical analysis. GB contributed to imaging concerning the patient. IJ wrote the first draft of the manuscript. CH, GB, MK, and FW wrote sections of the manuscript. All authors contributed to manuscript revision, read, and approved the submitted version.

## Conflict of interest

The authors declare that the research was conducted in the absence of any commercial or financial relationships that could be construed as a potential conflict of interest.

## Publisher's note

All claims expressed in this article are solely those of the authors and do not necessarily represent those of their affiliated organizations, or those of the publisher, the editors and the reviewers. Any product that may be evaluated in this article, or claim that may be made by its manufacturer, is not guaranteed or endorsed by the publisher.
